# We are like that only

**DOI:** 10.4103/0971-3026.45334

**Published:** 2009-02

**Authors:** Bhavin Jankharia

**Affiliations:** Editor-in-Chief, The Indian Journal of Radiology & Imaging, Bhaveshwar Vihar, Sardar V P road, Mumbai - 400 004. Email: editor@ijri.org

As Dr. Kalyanpur mentions in his commentary on the article titled “Teleradiology: an Indian perspective”,[1] the majority of us have no concept of quality and commitment.

He goes on to describe the current situation where a large number of so-called teleradiology providers in the country have sprung up to take advantage of the radiology outsourcing boom, but land up offering suspect quality reports, without any audits or conformity to International standards. This is hurting our reputation as an outsourcing hub.

In the same vein, commitment is as big an issue.

Recently, I was asked to contribute a review article, for a special issue of an indexed Indian journal, along with a co-author. Due to a host of unfortunate circumstances, I was unable to meet the deadline and in fact could submit the article only two months later, in the bargain, completely stressing out the guest editor, who has now sworn never to guest edit a special issue again. Over and over again, she kept wondering how my editorial team and I manage, considering that of the twelve authors she asked for contributions from, six foreign and only one Indian author submitted in time. She had to waste a lot of time, chasing down the other five Indian authors (one of them being me) and even after that, many of the submissions were not upto the mark or did not adhere to the guidelines for submission.

At the IJRI, we have similar problems with all our mini-symposia. All our invited foreign authors always submit on time. Barring two exceptions, none of our invited Indian authors have ever submitted on time.

I believe that the reason for this is our famous inability to say “no” and the fact that those who are good are quite often over-committed. I would like to use this forum to appeal to all guest authors to please stick to time schedules, so that the issues can be prepared in time. Else, the stress levels that the guest editor and the rest of us have to endure are sometimes just not worth all this.

In this issue, we are carrying the 2^nd^ part of the obstetric mini-symposium. The next two issues will have a mini-symposium on Breast Imaging. We are also starting a series of articles on tuberculosis, by experts in the field.

Wish you all a very Happy New Year.
